# A large amount of microscopic precipitates are inevitably injected during infusion therapy without an in-line filter

**DOI:** 10.1093/omcr/omab134

**Published:** 2022-02-19

**Authors:** Shinya Shimoyama, Daisuke Takahashi, Syuhei Arai, Yuji Asami, Kimiko Nakajima, Kentaro Ikeda, Takumi Takizawa, Tomio Kobayashi

**Affiliations:** Department of Pediatric Cardiology, Gunma Children’s Medical Center, Shibukawa 377-8577, Japan; Department of Pharmacy, Gunma Children’s Medical Center, Shibukawa 377-8577, Japan; Department of Pediatric Cardiology, Gunma Children’s Medical Center, Shibukawa 377-8577, Japan; Department of Pediatric Cardiology, Gunma Children’s Medical Center, Shibukawa 377-8577, Japan; Department of Pediatric Cardiology, Gunma Children’s Medical Center, Shibukawa 377-8577, Japan; Department of Pediatric Cardiology, Gunma Children’s Medical Center, Shibukawa 377-8577, Japan; Department of Pediatrics, Graduate School of Medicine, Gunma University, Maebashi 371-8511, Japan; Department of Pediatric Cardiology, Gunma Children’s Medical Center, Shibukawa 377-8577, Japan

## Abstract

Infusion route problems can have a significant impact on hemodynamics in children with severe heart failure. Here, we report the case of a 13-year-old girl with dilated cardiomyopathy. Her condition fluctuated due to frequent occlusion of the central venous catheter (CVC) route. However, a quick check revealed no apparent abnormalities in the CVC, infusion route, in-line filter or infusion pump. Scanning electron microscopy revealed that dobutamine and heparin had crystallized and that the in-line filter membrane was occluded. This case emphasizes the importance of proper infusion route management in pediatric patients with severe heart failure. Even drugs that are used daily may form microscopic crystals at several concentrations and administration rates. Without an in-line filter, microscopic particles are injected into the body, and there is no evidence that the injected crystals do not cause permanent damage.

## INTRODUCTION

Microscopic particles are infused with a visible precipitate in the infusion circuit during infusion therapy [1]. These fine particles are formed by drug–drug interactions, which may cause multiple organ dysfunction [2]. On the other hand, we encountered an occlusion in the infusion route that interrupted therapeutic drug administration. We showed that invisible particles are infused without a visible precipitate using an in-line filter. We report a case that indirectly demonstrates the presence of microscopic crystals on an in-line filter membrane.

## CASE REPORT

**Figure 1:**
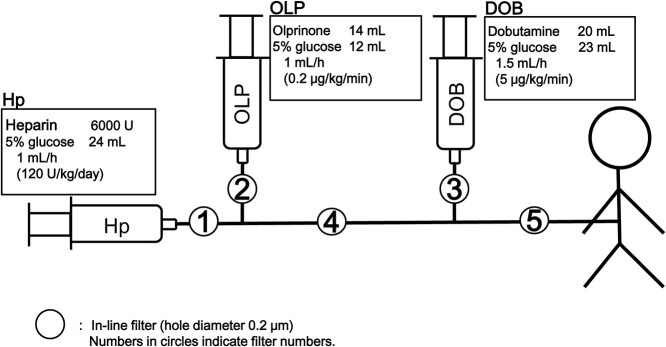
Schematic representation of the infusion line used in this case. The infusion fluid (5% glucose with heparin [Hp], olplinone [OLP] and dobutamine [DOB]) was administered through this line (no other drugs were administered) and an in-line filter with a pore size of 0.22 μm (Neo96; Pall Co., Ltd., Port Washington, NY) was used. No stopcock set was used.

We report a case of a 13-year-old girl with dilated cardiomyopathy presenting with severe cough. Because of poor cardiac contraction, she was admitted to the intensive care unit and treatment included catecholamine, olprinone, diuretics, vasodilators and heparin. She improved gradually but drug infusion administration was repeatedly interrupted due to route obstruction. We infused dobutamine, olprinone and heparin diluted with 5% glucose solution through the route using an in-line filter with a pore size of 0.22 μm (ref. Neo96; Pall Co., Ltd., Port Washington, NY) (Fig. 1), and no other drug was administered through the same line. We investigated the cause of frequent obstruction in the occluded route using unused routes as controls and measured the following four indices: (1) visual observation of the filter on the membrane surface and residual liquid in the filters; (2) residual liquid properties (such as pH of the residual liquid upstream and downstream of the filter), presence of turbidity and insoluble substances, and filter flow rate test; (3) filter membranes were examined using a scanning electron microscope (SEM) after vacuum vapor deposition with platinum and palladium and (4) elemental analysis of precipitates on the membrane surface found by SEM using an energy dispersive X-ray (EDX) analyzer.

Visual observation revealed that the entire filter circuit was filled with a colorless and transparent liquid, and no abnormality was found upstream or downstream in the filter or route. The flow rate tests showed that only the final filter (filter 5) showed a marked decrease (17%) when the flow rate of the unused filter was set to 100% (Table 1).

**Table 1:** Comparison of visual observation, filter flow rate test and residual liquid properties. After filling the filter circuit with 500 mL distilled water, the distance from the liquid level of the bag to the filter was set at 92 cm, and the volumes of water that passed through the filters were measured, respectively.

No. of filter	No. 1	No. 2	No. 3	No. 4	No. 5
Surface of the filter	np	np	np	np	np
Captured material	(−)	(−)	(−)	(−)	(−)
pH of the residual liquid (upstream/downstream)	5.7/5.7	3.5/3.5	5.2/5.4	4.4/4.8	4.7/4.5
Residual liquid color (upstream/downstream)	TP/TP	TP/TP	TP/TP	TP/TP	TP/TP
Filter flow rate (%)^a^	70%	74%	74%	72%	17%
Degree of flow rate decrease	mild	mild	mild	mild	severe

^a^The flow rate of unused products was set to 100%, TP: transparent, No: number, np: no precipitate.

**Figure 2:**
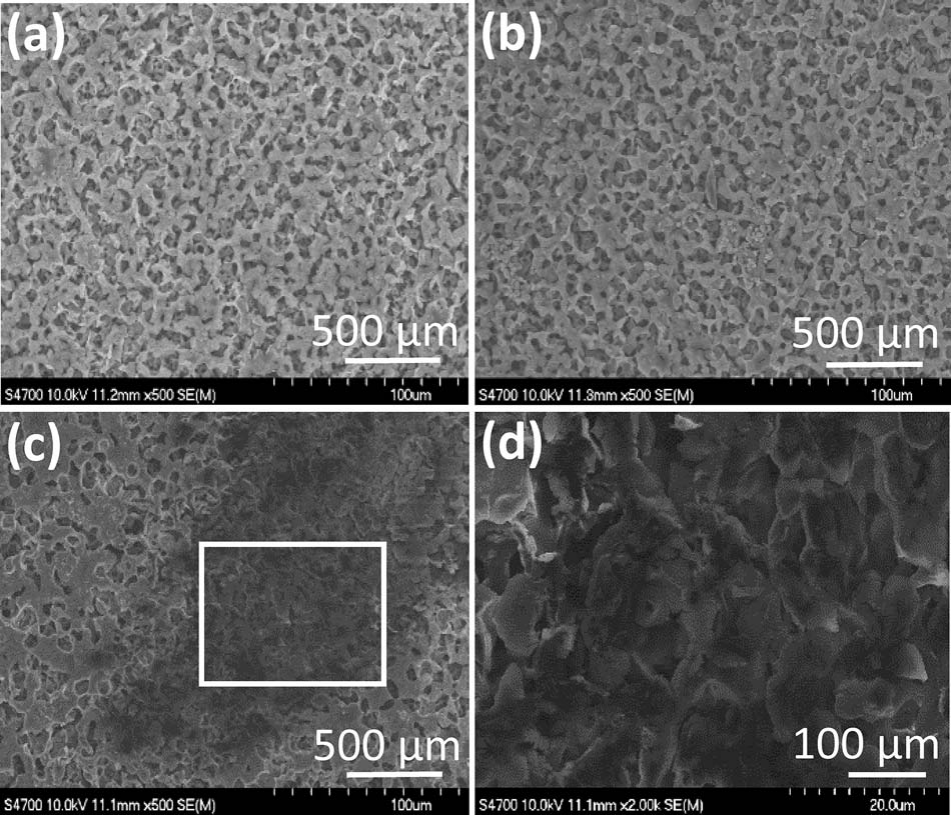
Upstream side surface of filter membranes observed under a scanning electron microscope. (**a**) The surface of the membrane of the unused filter (control) shows no deposits on the membrane. (**b**) No significant deposits on the filter membranes can be seen on filters 1–4. The photo shows filter 1. Filters 2–4 produced the same results as filter 1 and were, therefore, omitted. (**c**) Precipitates on the surface of filter 5. (**d**) Photo of filter 5 taken by enlarging the area surrounded by the white line on image C.

**Figure 3:**
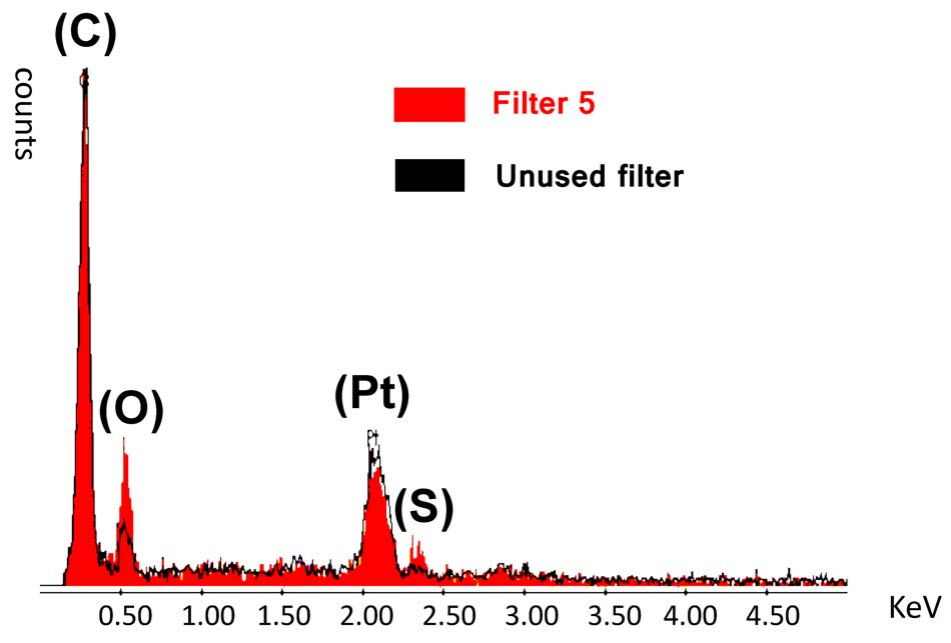
Surface comparison by energy dispersive X-ray analysis between filter 5 and an unused filter. The area under the red curve shows the composition of the substances that were captured on filter 5, and the area under the black curve is the control. The peaks of four elements (carbon, oxygen, platinum and sulfur) are observed. After vapor deposition of platinum and palladium on the filter membrane and pretreatment, scanning electron microscopy was performed. KeV: kilo-electron-volt.

**Figure 4:**
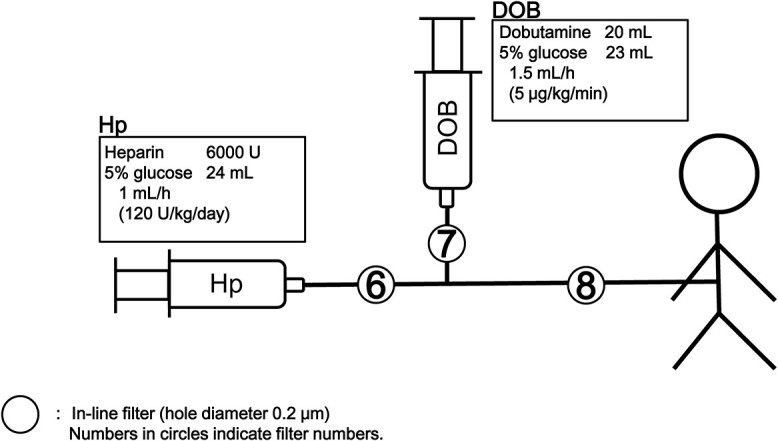
Schematic representation of the infusion line used in additional experimental tests. The infusion fluid (5% glucose with heparin [Hp], and dobutamine [DOB]) was infused through this line (no other drugs were administered), and an in-line filter with a pore size of 0.22 μm (Neo96; Pall Co., Ltd., Port Washington, NY) was used. No stopcock set was used.

SEM observations showed no significant deposits on the used filter membranes, except for filter 5, and no significant deposits on the filter membranes of the controls (Fig. 2a and b). A large amount of trapped material was confirmed on the filter surface on the upstream side, and solid material was observed on the filter surface on the downstream side of filter 5 (Fig. 2c and d). Elemental analysis of the precipitates found by SEM on the upstream side of filter 5 was performed using the EDX in filter 5 (Fig. 3). The area under the red curve shows the compositions of the substances captured in filter 5, and the area under the black curve is the control; more oxygen and sulfur were present in the precipitate found in filter 5 than in the unused filters. Additional experimental tests were performed with heparin and dobutamine to identify the causative agent of crystal formation (Fig. 4). Filter 8 displayed a marked decrease in flow rate (Table 2).

## DISCUSSION

We showed that many microscopic particles were formed in the infusion circuit during drug infusion therapy and that these particles can be infused into the body during infusion therapies without in-line filters. In-line filters are effective against foreign substances, such as glass fragments, air and microorganisms [3, 4]. Recently, a large number of drug particles were detected in infusion routes. These particles were identified using particle counters [1, 5]. Perez *et al*. [1] reported a visible precipitate in the infusion line, but in our case, no apparent drug precipitates were visible to the naked eye in the lines or filters. However, we confirmed the presence of microscopic precipitates after performing flow rate tests and SEM. In SEM and EDX analyses, oxygen and sulfur were found to be significantly higher in filter 5 than in the control, suggesting that the trapped materials contained organic matter derived from the infused drugs. This indicates that the therapeutic drug crystallized and that the filter was blocked by microscopic precipitates. Heparin and dobutamine could change when combined, which may depend on the concentration of the drug solution and the contact time between the drugs [6, 7]. Therefore, we must always consider the possibility that precipitates will form under various conditions, even in drugs that are used daily. Drug–drug interactions may not be readily apparent in clinical practice.

It is known that crystals injected into the body can be transported to organs and pose a risk of multiple organ failure [8, 9]. Children are considered to be at high risk for developing this condition [5]. The organs of patients with congenital heart disease (CHD) are more likely to be

affected because they have a right–left short circuit. Furthermore, low-weight CHD patients are treated with high concentrations of drugs that are infused at a low velocity to promote a reaction between the drugs. Nishikawa *et al*. [10] reported that SEM confirmed the presence of bacteria adhering to the drug precipitate on the surface of the lumen of the CVC. We speculate that the use of in-line filters may prevent the influx of precipitates that trigger bacterial infections and reduce the risk of infection. Further studies and careful line management are needed to prevent the harmful influx of fine particles.

A limitation of our approach is the impossibility of evaluating all possible combinations of the drug solution’s concentration and infusion velocity. This is because in children with severe heart disease, the amount of permitted fluid intake varies greatly. However, we have confirmed reproducibility at this concentration.

**Table 2:** Comparison of the visual observation findings, filter flow rate test results, and residual liquid properties in the additional experimental tests. After filling the filter circuit with 500 mL distilled water, the distance from the liquid level of the bag to the filter was set at 92 cm, and the volumes of water that passed through the filters were measured, respectively

No. of filter	No. 6	No. 7	No. 8
Surface of the filter	np	np	np
Captured material	(−)	(−)	(−)
pH of the residual liquid (upstream/downstream)	5.2/5.2	6.0/6.1	5.5/5.4
Residual liquid color (upstream/downstream)	TP/TP	TP/TP	TP/TP
Filter flow rate (%)^a^	81%	82%	12%
Degree of flow rate decrease	mild	mild	severe

^a^The flow rate of unused products was set to 100%. TP: transparent, No: number, np: no precipitate.

In conclusion, clinicians should be aware of the risk of precipitate formation and reconsider the prescription when starting drug infusion therapy. The long-term effects of injected crystals are largely unknown, and in-line filters provide a practical option to reduce potential risks.
